# 2^nd^ coordination sphere controlled electron transfer of iron hangman complexes on electrodes probed by surface enhanced vibrational spectroscopy†
†Electronic supplementary information (ESI) available: Details on data treatment procedure for TR-SERR and SEIRA spectroscopy and electrocatalysis. See DOI: 10.1039/c5sc02560e


**DOI:** 10.1039/c5sc02560e

**Published:** 2015-09-07

**Authors:** H. K. Ly, P. Wrzolek, N. Heidary, R. Götz, M. Horch, J. Kozuch, M. Schwalbe, I. M. Weidinger

**Affiliations:** a Department of Chemistry , Technische Universität Berlin , PC14, Straße des 17. Juni 135 , D-10623 Berlin , Germany . Email: inez.weidinger@tu-berlin.de ; Email: khoaly@mailbox.tu-berlin.de; b Department of Chemistry , Humboldt Universität zu Berlin , Brook-Taylor-Str. 2 , D-12489 Berlin , Germany . Email: matthias.schwalbe@hu-berlin.de

## Abstract

Iron hangman complexes exhibit improved catalytic properties regarding O_2_ and H_2_O_2_ reduction, which are attributed to the presence of a proton donating group in defined vicinity of the catalytic metal centre. Surface enhanced resonance Raman (SERR) and IR (SEIRA) spectro-electrochemistry has been applied concomitantly for the first time to analyse such iron hangman porphyrin complexes attached to electrodes in aqueous solution. While the SERR spectra yield information about the redox state of the central iron, the SEIRA spectra show protonation and deprotonation events of the 2^nd^ coordination sphere. To investigate the influence of a proton active hanging group on the heterogeneous electron transfer between the iron porphyrin and the electrode, two hangman complexes with either an acid or ester functional group were compared. Using time resolved SERR spectroscopy the electron transfer rates of both complexes were determined. Complexes with an acid group showed a slow electron transfer rate at neutral pH that increased significantly at pH 4, while complexes with an ester group exhibited a much faster, but pH independent rate. SEIRA measurements were able to determine directly for the first time a p*K*_a_ value of 3.4 of a carboxylic hanging group in the immobilized state that shifted to 5.2 in D_2_O buffer solution. The kinetic data showed an increase of the heterogeneous electron transfer rate with the protonation degree of the acid groups. From these results, we propose a PCET which is strongly modulated by the protonation state of the acid hanging group *via* hydrogen bond interactions.

## Abstract 2

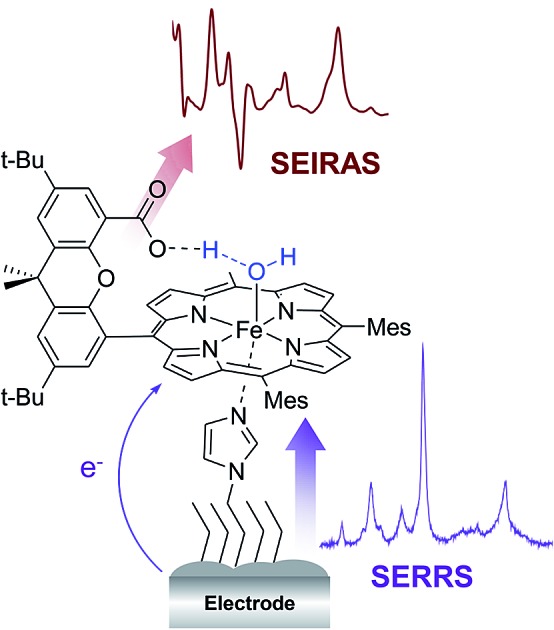
Surface enhanced vibrational spectroscopy shows the correlation between electron transfer kinetics and protonation degree of Fe Hangman complexes on electrodes.

## Introduction

Second coordination sphere assisted reactions are crucial for the efficiency of numerous catalytic transformations. For example, in nature a variety of different reactions are catalysed by heme cofactors where selectivity of the reaction is induced by acidic or basic amino acids in the coordination sphere around the heme environment.^1^–^3^ This highly ordered arrangement of proton donating or accepting groups defines the catalysed reaction by the heme group, which can range from reduction of hydrogen peroxide to water (catalase), substrate oxidation (peroxidase), binding of molecular oxygen (myoglobin, hemoglobin), oxygen reduction to water (cytochrome c oxidase) or hydroxylation of different compounds (cytochrome P450).

Synthetic biomimetic molecular catalysts copy the essence of the reaction centres of their enzymatic analogues, and exploit the optimally evolved active structures for maximal performance.^4^,^5^ In this respect, they exhibit numerous advantages compared to their biological idols. On the one hand, due to their smaller size, better substrate accessibility and higher stability, these compounds bear a high potential to be used in technological applications such as biomimetic fuel cells. On the other hand, the study of molecular catalysts is highly valuable in general. Well-defined synthetic catalysts allow detailed investigations of the catalytic mechanism at a molecular level and precise fine-tuning of desired catalytic activity using synthetic chemistry. Understanding the structure–function relationships of catalytically active sites can in turn enhance the knowledge of biological catalysis.

One of the challenges in catalyst design is to mimic the electron/proton transfer interplay that has been naturally optimized in enzymatic catalysis. In this regard, hangman porphyrin complexes that carry a heme group and an arbitrary functional “hanging” group positioned in a defined distance to the reaction centre, constitute an interesting model system to study the influence of the 2^nd^ coordination sphere.^6^–^8^ Hangman complexes that exhibit a hanging carboxylic acid group have been shown to significantly enhance the catalase^9^ and oxidase reaction in solution in comparison to complexes with non-acidic hanging groups.^10^,^11^ For similar iron hangman corroles, a catalase like reaction mechanism has been proposed that involves the carboxylic acid group as a proton donor site.^12^ Cobalt hangman porphyrins and corroles have also been successfully tested in electrocatalytic dioxygen reduction and hydrogen evolution.^13^–^15^ For a technological application in fuel cells, the hangman complexes have to be immobilized on an electrode surface. In contrast to homogeneous reactions, the adsorption provides numerous advantages such as site isolation of catalytically active centres, facilitated catalyst recycling and the general use of aqueous solvents.^16^–^19^ Importantly, the created direct electronic contact can lead to enhanced electron transfer (ET) between catalyst and electrode.^20^ ET processes play a crucial role in the electrocatalytic mechanism as reaction intermediates are generated through electron acceptance/donation. Therefore, the rate of this process may not only determine overall catalytic activity but has also been shown to directly influence the reaction products in case of oxygen reduction.^21^

The study of adsorbed compounds is challenging and requires adaptation of suitable spectroscopic methods that provide structural insights into the catalytic processes at the surface. The elucidation of these heterogeneous reactions is a major prerequisite for promoting technological application of hangman compounds. In this regard, surface enhanced vibrational spectroscopy has the surface sensitivity to investigate sub-monolayer concentrations of immobilized molecules. In particular, the two vibrational spectroscopic techniques, surface enhanced Raman (SER) spectroscopy and surface enhanced infrared absorption (SEIRA), are able to provide different and often complementary information at a molecular level that can be used to monitor both, redox changes and protonation events of adsorbed compounds. Particularly, for heme containing molecules, laser excitation with violet light allows exploitation of the molecular resonance effect yielding surface enhanced resonance Raman (SERR) spectroscopy to selectively monitor the vibrational modes of the absorbing porphyrin ring. Hence, SERR spectro-electrochemistry has been used extensively in the past, in particular, to analyse the redox and catalytic properties of surface bound heme enzymes.^22^–^26^ Recently, also SERR measurements of surface bound heme containing molecular catalysts were presented providing interesting insights into their catalytic mechanism by *inter alia* monitoring direct product transformation at the heme using a RDE-SERR setup.^27^–^32^ SEIRA spectroscopy on the other hand monitors all vibrations of the surface bound molecules. It is, however, especially sensitive to polar vibrations, such as carboxylic acid groups, and has been used in the past *e.g.* to analyse the protonation of a single glutamic acid residue in a complex protein matrix.^33^ The combination of both types of surface enhanced vibrational spectroscopies has been applied to understand the effect of protein reorientation in enzymatic electrocatalysis.^34^ In the present work it is used for the first time to study small electrocatalytic active complexes on surfaces and to correlate electron transfer with proton delivery events in the coordination sphere. Thus, this technique is able to provide unique insight into the 2^nd^ coordination sphere controlled heterogeneous electron transfer (HET) of molecular catalysts on surfaces *in operando*. In this paper, we present the first results regarding electron and proton transfer processes of surface bound heme based hangman complexes in the absence of substrate using SERR and SEIRA spectroscopy.

## Materials and methods

Iron hangman porphyrin compounds were synthesised according to published procedures.^6^,^11^ Briefly, the free base porphyrins POH and POMe were synthesised as described in ref. 6 and reacted with iron(ii) chloride in dimethylformamide as described in ref. 11. Aerobic acid workup yields in the formation of the corresponding chloroiron(iii) porphyrin complexes.^11^

For SERR measurements, an electrochemically roughened Ag ring electrode was used as solid support prepared by a previously described procedure.^35^ For SEIRA measurements, a Si prism was coated chemically with a thin Au layer that was used as electrode interface. A detailed description of the process and measurement geometry can be found here.^36^ The respective electrodes were incubated overnight (>16 h) in an ethanolic solution containing 0.6 mM and 0.3 mM of 1-heptanethiol (98%, Sigma Aldrich) and 1-(11-mercaptoundecyl)imidazole (96%, Sigma Aldrich), respectively. This procedure leads to the formation of a mixed self-assembled monolayer (SAM) on top of the electrode's surface. The electrodes were cleaned with abundant ethanol prior to use. Hangman adsorption was achieved by incubation of the SAM coated electrodes with a *ca.* 10 μM solution of the hangman compound in DCM.^28^ Immobilisation was finished after 2 h, and unspecific bound, *i.e.* physisorbed, compounds removed by rinsing with abundant DCM (>99.8%, Sigma Aldrich).^28^

The electrodes were subsequently mounted into a homemade spectro-electrochemical cell prepared for potential controlled SERR experiments and rotated (10 Hz) during measurements to avoid laser induced degradation. Rotation of the electrode is further necessary to minimize diffusion limitation of substrate or protons.^37^ SEIRA measurements were carried out using a home-built spectro-electrochemical cell in the ATR mode in Kretschmann geometry using the Si prism as waveguide.^36^ For measurements in aqueous phosphate buffer (PBS) solution, an Ag/AgCl 3 M KCl reference electrode was used (DriRef, WPI). Unless otherwise mentioned, PBS buffer always refers to pH 7 and 100 mM concentration. Catalysis tests were performed using diluted H_2_O_2_ (30% in water, Sigma Aldrich) in buffer using a commercial rotating Au disc electrode setup (Pine Instruments). All employed solvents and chemicals were purchased and used without further purification. All experiments were performed under Ar atmosphere.

SERR spectra were acquired using the 413 nm line of a krypton ion laser (Coherent Innova 300c) coupled to confocal Raman setup with a single-stage spectrograph (Jobin Yvon LabRam 800 HR) equipped with a liquid-nitrogen-cooled CCD detector in 180° back scattering geometry. The laser light was focused using a Nikon 20× objective (N. A. 0.35) with a working distance of 20 mm. Laser power on the sample was about 1 mW. Spectra acquisition times varied from 5 to 60 s for stationary and from 120 to 180 s for time resolved measurements, respectively. All experiments were repeated several times to ensure reproducibility. For time resolved (TR) SERR experiments, potential jumps of variable height and duration were applied to trigger the redox reaction as previously described.^38^ The SERR spectra were measured at different delay times following the potential jump using synchronized laser light modulators. After background subtraction the spectra were treated by component analysis, in which the spectra of individual species, *i.e.* components, were fitted to the measured spectra using a home-made analysis software.^39^ SEIRA measurements were carried out using a Bruker IFS 66v/s spectrometer equipped with a photoconductive MCT detector. 400 scans were co-added for a spectrum with a final resolution of about 4 cm^–1^.

## Results and discussion

**Fig. 1:**
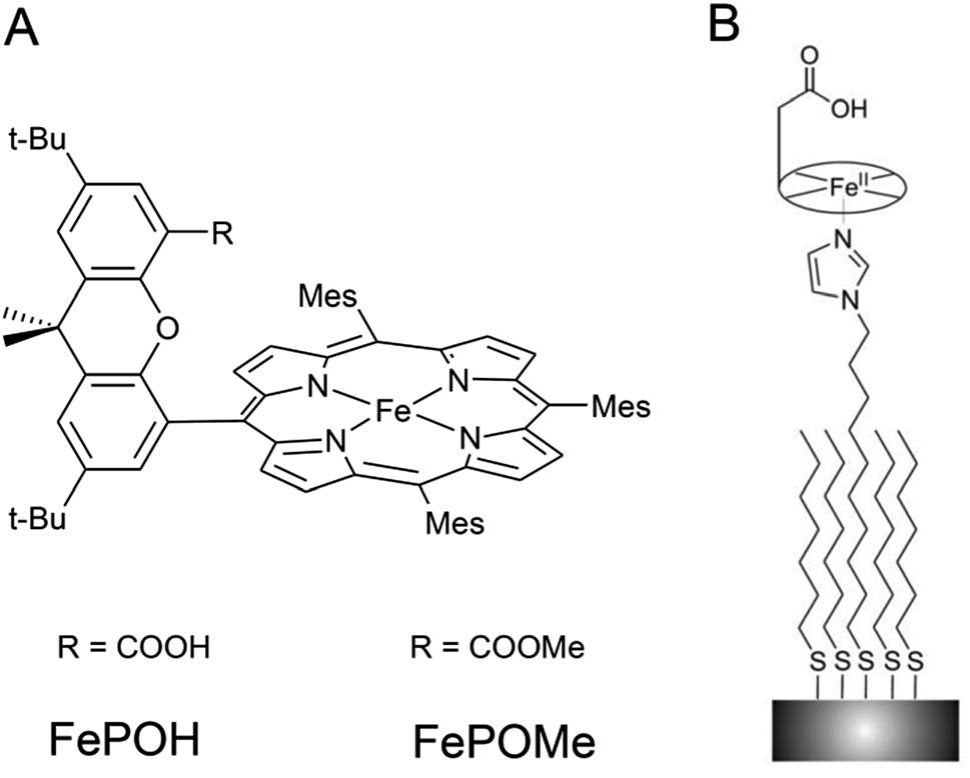
(A) Structure of FePOH and FePOMe hangman complexes. (B) Schematic representation of the hangman immobilization on electrodes using a mixed imidazole terminated SAM.

Two iron hangman porphyrin complexes with a different hanging functional group were synthesized according to published procedures.^6^,^11^ In the first complex, the hanging group consists of a proton active carboxylic acid terminus while the second exhibits a carboxylic ester group (see Fig. 1). The former complex is abbreviated as FePOH, the latter as FePOMe. Immobilization of the hangman complexes on SERR active Ag supports was achieved by coating the supports with a mixed monolayer following a recently published procedure.^28^ The monolayer consists of two types of molecules: a shorter methyl terminated (HS–(CH_2_)_6_–CH_3_) and a longer imidazole terminated (HS–(CH_2_)_10_–Im) alkanethiol. Specific binding of the hangman compound is expected to occur by coordination of the imidazole nitrogen to the heme iron as present in heme–histidine systems of biological heme enzymes. Unspecific bound compounds were removed by rinsing with abundant dichloromethane (DCM). Subsequently, the solution was either changed to acetonitrile (ACN) or PBS buffer. As a first step, it was checked whether the hangman complexes could preserve their catalase function upon immobilization. For this, catalytic catalase activity of the immobilized hangman complexes towards H_2_O_2_ oxidation was detected by chronoamperometry. A rotating Au electrode coated with SAM and hangman complexes was immersed into a 100 mM PBS pH 7 solution and the potential set to +0.1 V. Upon stepwise H_2_O_2_ substrate addition, increasing catalytic currents were observed confirming catalytic activity in the immobilized state in aqueous PBS buffer solution (for details see ESI Section 1†). For the concentration of H_2_O_2_ at half maximum current, a value of 8 ± 4 mM and 3 ± 1 mM H_2_O_2_ was determined for FePOH and FePOMe, respectively. Moreover, a *ca.* threefold higher catalytic current was observed for FePOH than for FePOMe under identical experimental conditions (ESI Fig. S1†).

### SERR spectroscopy of immobilised hangman complexes

**Fig. 2:**
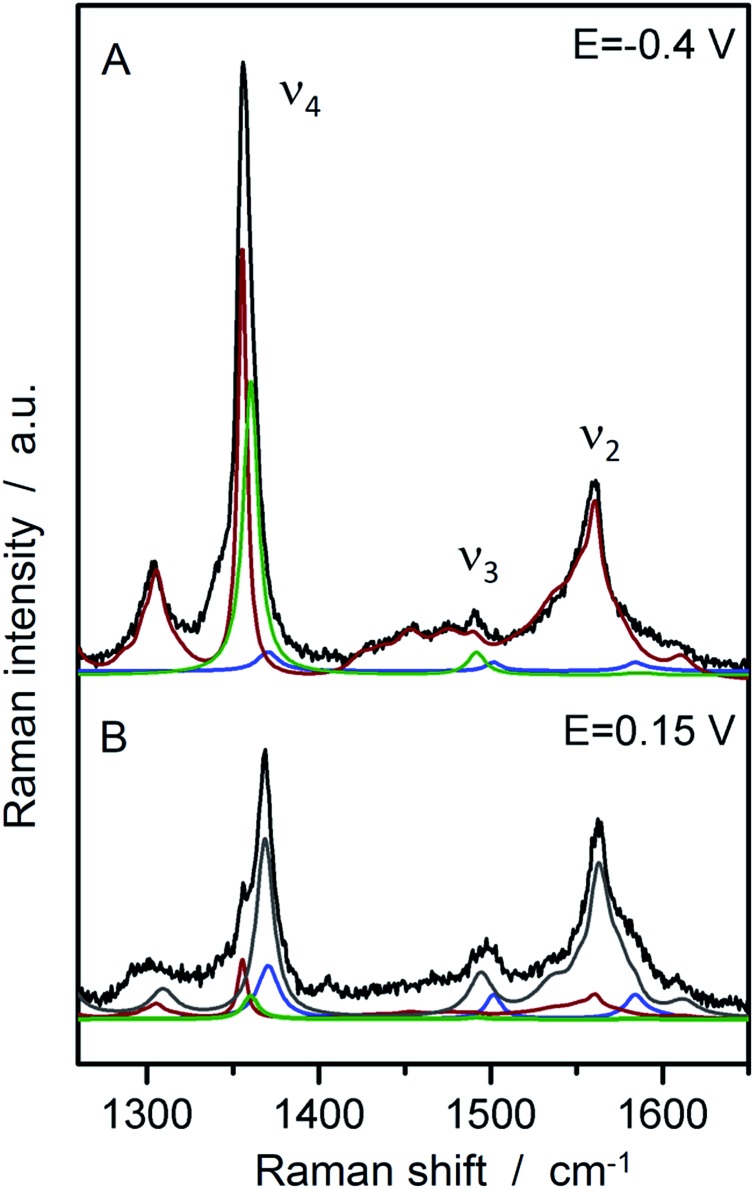
SERR spectra of FePOH in 100 mM PBS buffer at –0.4 V and +0.15 V *vs.* Ag/AgCl 3 M with the respective component spectra: HS red (red), HS ox (grey), LS red (green), LS ox (blue).

In a second step, SERR spectroscopy was performed on the hangman/electrode system using a rotating Ag ring electrode. Intense SERR spectra of the immobilised hangman compounds were obtained upon 413 nm Soret laser excitation. SERR spectra recorded in the absence of the imidazole terminated alkanethiol linker molecule afforded no SERR signals (ESI Fig. S2†) supporting the proposed direct binding of the heme iron to the imidazole (see ESI section 2† for more information). Fig. 2 shows the SERR spectra of the imidazole immobilised FePOH hangman complex on Ag electrodes at –0.4 V and 0.15 V applied electrode potential in Ar purged PBS buffer. The spectra resemble typical spectra of heme compounds, exhibiting strong marker bands around 1370 cm^–1^ (*ν*_4_) and 1575 cm^–1^ (*ν*_2_) as well as a broader band with lower intensity at around 1495–1500 cm^–1^ (*ν*_3_). These bands are indicative for the redox, spin and ligation state of respective heme compounds.^40^ To extract the contribution of the different redox and configurational species, a component fit analysis was performed (for details of the fitting procedure see section 3 in ESI†).^39^ Briefly, known component spectra of different heme types were used and modified accordingly. As a result, the presence of two heme spin states (high spin (HS) and low spin (LS)) were found, each appearing in two different redox states (Fe^III^ and Fe^II^). The molecular nature of the different (spin) species is not known *a priori*. However, regarding the surface functionality of the SAM, we propose that the HS species is represented by an iron complex with the imidazole group of the SAM as fifth axial ligand. A water or hydroxide molecule loosely attached to the heme iron as a sixth ligand is very likely and usually does not lead to a change in spin state (*vide infra*).^40^ To induce the observed LS state, a stronger binding sixth ligand is required. The nature of this 6^th^ ligand is yet unknown and it might be a residue of the synthesis procedure or an unwanted side-product that is formed on the electrode surface. This species furthermore shows only a limited redox activity (*vide infra*). For the following data evaluation, we therefore concentrate on the Fe–HS species.

**Fig. 3:**
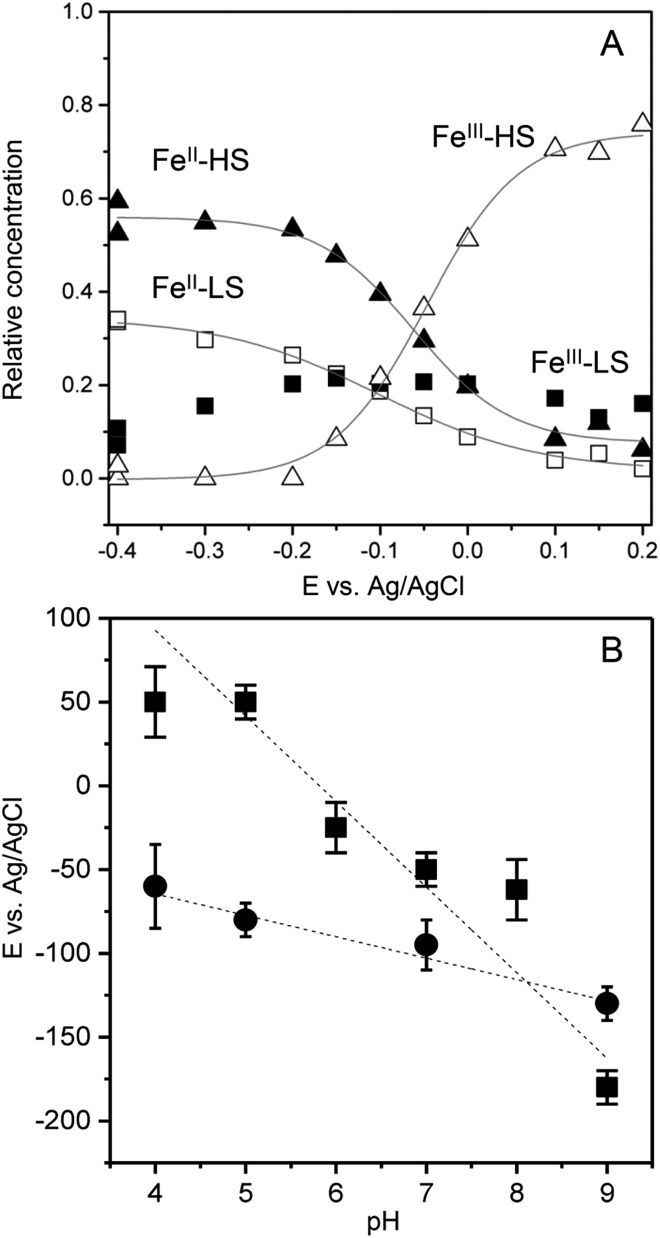
(A) Relative contributions of the FePOH component spectra (Fe^III^–HS: open triangles, Fe^II^–HS: solid triangles, Fe^III^–LS: solid squares, Fe^II^–HS: open squares) as a function of electrode potential. (B) Redox potentials of FePOH (squares) and FePOMe (circles) as a function of pH.

To transform SERR intensities into relative surface concentrations, spectral intensities of the different heme species, determined from the component analysis, were multiplied with respective SERR cross sections accounting for the different RR scattering efficiency and summed up to a total intensity.^40^–^42^ Relative concentration of a particular heme species was derived by calculating the relative spectral intensity of this species in the overall intensity. Calculation and determination of the cross sections followed established procedures for heme proteins (for details see section 4 in the ESI†).^40^–^42^ The relative surface concentrations of each species are shown in Fig. 3A as a function of applied potential. At the starting potential of 0.15 V, a mixture of oxidized HS and LS species is observed with the HS species as the major fraction. A reduced species that contains both, a LS and a HS conformation, arises at more negative potentials at the expense of the oxidized HS species. In contrast, the concentration of the oxidized LS species seems to be largely independent from the applied electrode potential.

A fit of the Nernst equation to the values of the oxidized HS species as a function of potential yields the redox potential *E*^0^ for the redox couple Fe^III^/Fe^II^–HS. The so derived values for *E*^0^ of FePOH and FePOMe are plotted in Fig. 3B as a function of pH. Here, significant differences are observed for the two types of hangman complexes. While FePOH shows a distinct dependence of *E*^0^ on pH, *E*^0^ of FePOMe remains almost pH independent. A linear fit of the data for FePOH yields a slope of –57 ± 5 mV pH^–1^.

### SEIRA spectroscopic probing of the hanging group

**Fig. 4:**
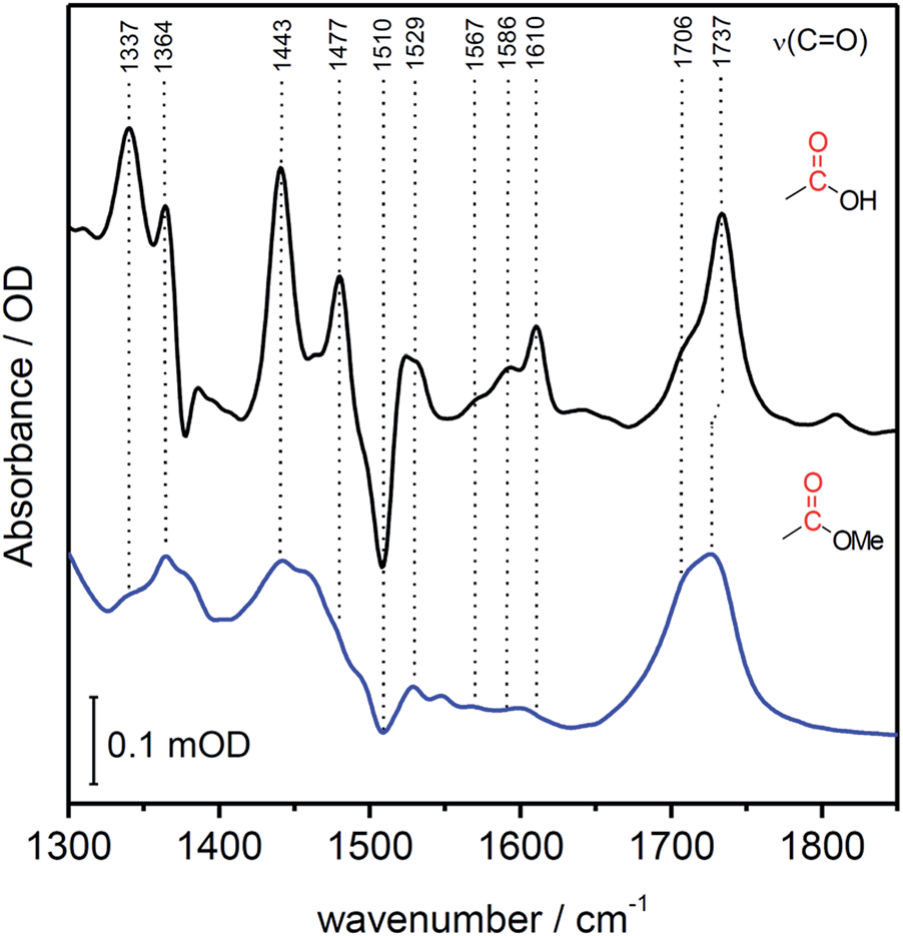
SEIRA spectrum of immobilized FePOH (black) and FePOMe (blue) in ACN. The SAM coated Au electrode was used as reference spectrum.

**Fig. 5:**
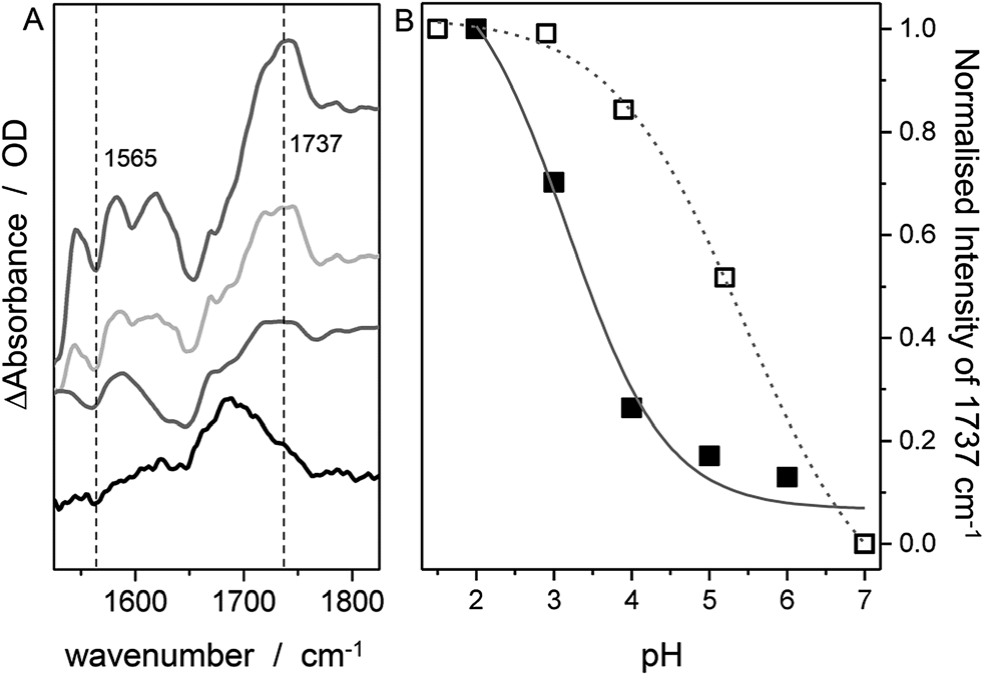
SEIRA difference spectrum of FePOH in PBS buffer of different pH. From bottom to top: pH = 5.5, 4.3, 3.0 and 1.6. The spectrum at pH 7 was used as reference. (B) Normalized intensity of the 1737 cm^–1^ band (or the 1715 cm^–1^ band in D_2_O) as a function of pH.

In contrast to SERR measurements, SEIRA experiments can visualize non-heme-related changes. For SEIRA measurements, the hangman complexes were immobilized in the same way as in the SERR experiment albeit in this case a nanostructured gold film deposited on a Si prism was used as electrode.^36^,^43^ First, adsorption of the SAM onto the electrode was followed. Here, a band pattern in the region from 3000 cm^–1^ to 2800 cm^–1^ and a band at 1113 cm^–1^ is observed (Fig. S3†). These bands can be attributed to modes with high contributions from C–H stretching vibration due to the methylene groups of the SAM molecules.^44^ Furthermore, a band at 1510 cm^–1^ is observed, which is assigned by comparison with literature and by DFT calculations to the *ν*(C=C) stretching mode of the deprotonated imidazole ring.^45^ A more detailed discussion on the band assignment is provided in section 2 of the ESI.† Fig. 4 shows the SEIRA spectrum of the immobilized FePOH and FePOMe compound in ACN solution using the SAM coated electrode as a reference. Upon addition of the compounds, the imidazole band at 1510 cm^–1^ disappears or shifts confirming that the hangman complexes indeed bind *via* the proposed coordinative Fe/N(imidazole) bond. Furthermore, for FePOH a prominent band arose at around 1737 cm^–1^ that can be assigned to the *ν*(C=O) stretching vibration of the protonated carboxylic acid of the hangman motif. In the case of FePOMe, this band is located at around 1727 cm^–1^ in accordance with an expected downshift for carbonyl stretching frequencies of esters with respect to acids.^44^ For FePOH and FePOMe, a shoulder at 1706 cm^–1^ is observed, which is more pronounced for FePOMe. As this band is observed for both complexes, we exclude that it originates from the carbonyl stretching vibration of the hanging group itself. More likely, the band may arise from a high shifted *ν*(C=N) vibration probably of the heme pyrrole or the imidazole C=N group. Interestingly, the *ν*(C=O) vibration of FePOH disappeared when the solution was changed to aqueous PBS buffer at pH 7. This observation can be explained with a deprotonation of the carboxylic acid group. Upon changing the pH of the buffer solution to low pH values, the band at 1737 cm^–1^ reappeared clearly associated with a decrease in intensity of the band at 1565 cm^–1^ as shown in Fig. 5A. This band most likely represents the asymmetric *ν*(COO^–^) stretching of the associated carboxylate base. The intensity of the 1737 cm^–1^ band was used to create a pH titration curve presented in Fig. 5B. From these measurements, we determine the p*K*_a_ value of the hanging acid group in aqueous solution to be 3.4 ± 0.2. Upon D_2_O exchange, the band of the FePOH shifts to 1715 cm^–1^ (ESI Fig. S7†). This constitutes a rather drastic downshift and might be caused by an additional overall change in the hydrogen/deuteron bonding network around the acid group. The p*K*_a_ of the acid group in D_2_O buffer was determined to be 5.2 ± 0.4 (Fig. 5B). Qualitatively such a shift in p*K*_a_ is in line with a predicted increase of basicity upon deuteration of carboxylic acids.^46^ Finally, SEIRA difference spectra were measured as a function of potential in ACN (with 10% MeOH) and PBS buffer. In both cases, no potential induced changes of the 1737 cm^–1^ band were observed (ESI Fig. S8†) indicating that the protonated/deprotonated form of the carboxylic hanging group is stable over the scanned potential range.

### TR-SERR spectroscopic determination of the HET rate

**Fig. 6:**
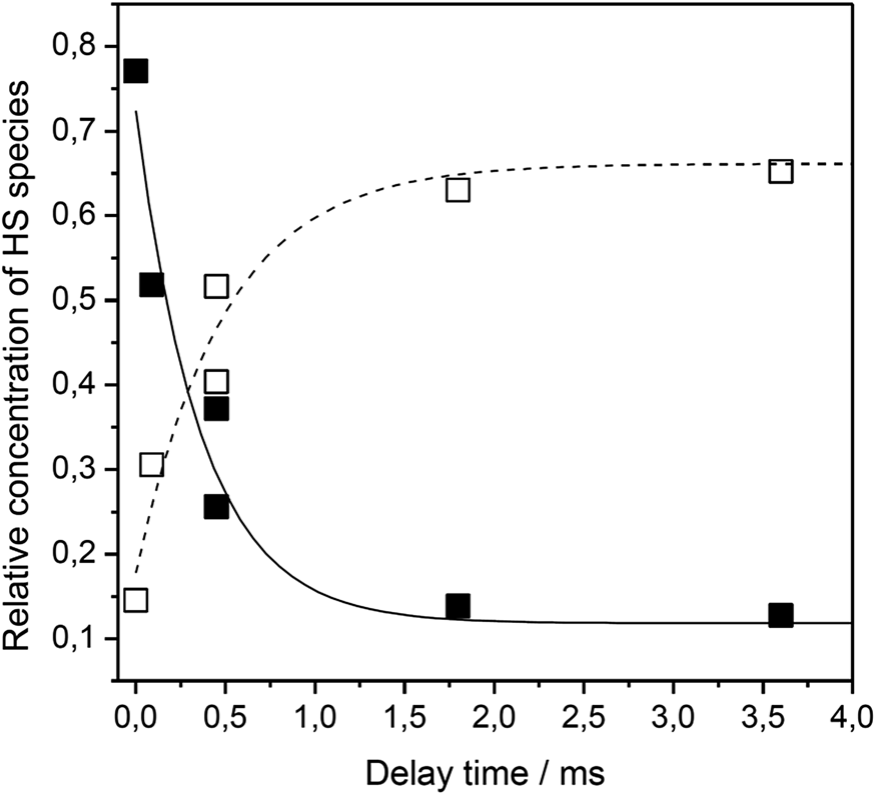
Relative contribution of the Fe^III^–HS (solid squares) and Fe^II^–HS (hollow squares) species of FePOH as a function of delay time after a potential jump from 0.15 V to 0.4 V.

Using time resolved SERR spectroscopy, the heterogeneous electron transfer rates *k*_HET_ of FePOH and FePOMe in aqueous and deuterated phosphate buffer solution were measured by following the oxidation state of the heme as a function of time.^38^ Measurements were performed at pH 7 and pH 4 to investigate the influence of the protonation state of the carboxylic acid group on the ET kinetics. The relative contribution of the oxidized Fe^III^–HS species was monitored as a function of delay time subsequent to a potential jump (see ESI section 6†). The initial potential was set to 0.10 V. The final potential was set in a way to yield an overpotential of *η* = *E* – *E*^0^ = –0.30 V taking into account the difference in redox potential at different pH values. For such a high overpotential, the rate observed for reduction can be set equal to the heterogeneous electron transfer rate *k*_HET_.^35^Fig. 6 shows a typical relaxation curve of the Fe^III^–HS species to the surface redox equilibrium at the final potential induced by the potential jump. In all measurements, we observed a fast initial drop of the Fe^III^–HS concentration that was sometimes followed by a much slower relaxation phase until equilibrium conditions were reached (ESI Fig. S11†). In order to achieve a consistent evaluation of the kinetic data, all kinetic traces were fitted with monoexponential functions. The rate constant is obtained by *k*_ET_ = 1/*τ* where *τ* is the time constant of the fit function (details on the TR SERR measurements are given in section 6 of the ESI†).^35^,^38^,^41^,^42^ The heterogeneous ET (HET) rate constants were obtained from several different experiments and their respective mean values are listed in Table 1. Due to scattering of the data, an average relative error of 15% for *k*_ET_ is stated. The determined HET rates obtained at significantly high overpotential show values in the order of several thousand per second. Among other factors, these fast ET rates might be a result of the direct wiring of the heme iron to the electrode affording a good electronic coupling and/or electron tunneling path.^20^ Again, a distinctly different behavior of FePOH compared to FePOMe was observed. While for FePOMe, the ET rates do not depend on the buffer pH within the given accuracy, the ET rate of FePOH is more than 25 times higher at pH 4 than at pH 7. A similar behavior is observed upon switching to deuterated phosphate buffer solution. For FePOMe, a slight decrease of the rate constant is observed upon changing from pH 7 to pH 4 in D_2_O. In contrast, the rates increased for FePOH by 6 times for the same set of measurements. Comparing the rates at the same pH in H_2_O *vs.* D_2_O, another remarkable observation is made. FePOMe shows almost no kinetic isotope effect (KIE) at both measured pH values. In contrast, the ET rates of FePOH at pH 7 increase more than tenfold affording, in fact, an inverse KIE = 0.08. A smaller inverse KIE = 0.3 is observed at pH 4.

**Table 1:** Electron transfer rates derived from TR SERRS in different buffer and pH.

Buffer	pH	*k*_HET_/s^–1^	
		FePOH	FePOMe
PBS H_2_O	7	100	7000
PBS H_2_O	4	2800	7800
PBS D_2_O	7	1300	7700
PBS D_2_O	4	8600	5600

### Mechanistic implications of the effect of the hanging group

**Scheme 1:**
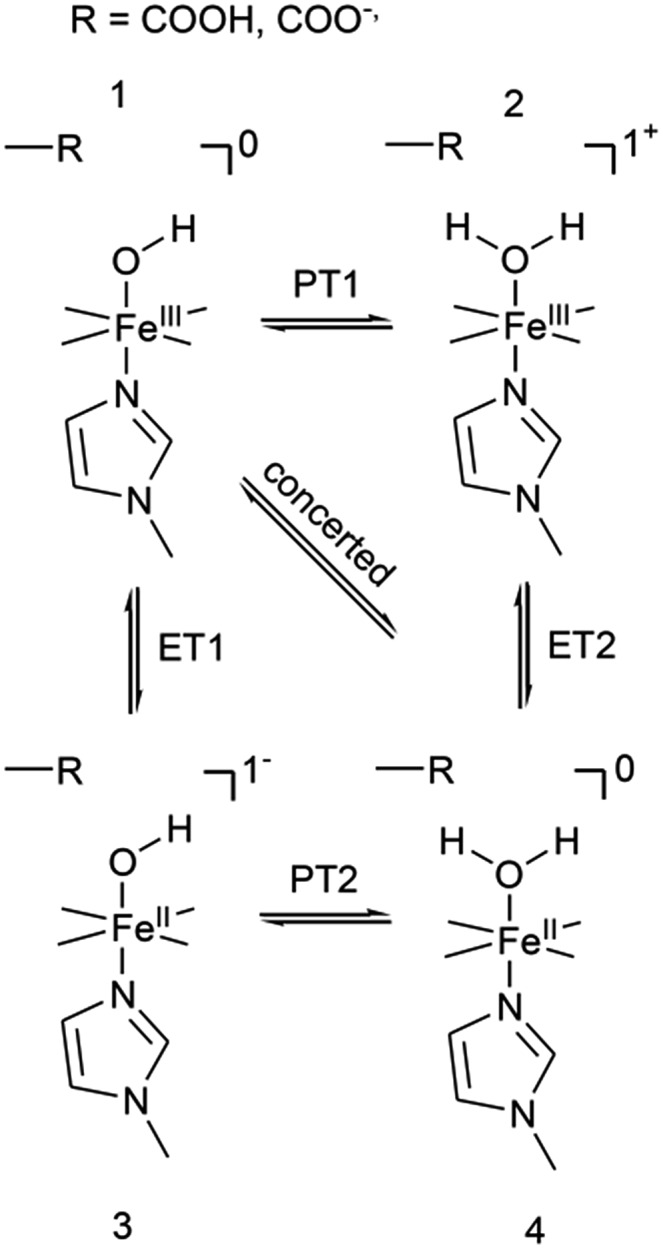
Proposed reaction scheme for a proton coupled ET reaction of the FePOH. The stated charge does not consider the hanging group.

The distinctly different behaviour of both compounds points to a direct perturbation of the redox thermodynamic and kinetic behaviour by the hanging group. While FePOMe shows almost no variation of *E*^0^ as a function of pH, FePOH exhibits a shift of *E*^0^ by –57 mV per pH unit. This finding strongly indicates a PCET step involved in the redox transition of FePOH from Fe^III^/Fe^II^. In this vein, the pH dependent shift further implies a transfer of one proton to the compound upon one electron reduction.^31^,^47^–^49^ A simple redox transition induced de-/protonation of the carboxylic group was not observed in potential dependent SEIRA experiments over a broad potential range and can therefore not account as accompanied PT process. More likely is the scenario of a water or hydroxyl ligand bound at the heme iron as 6^th^ ligand in the ferrous and ferric state, respectively. This additional protonable ligand may be able to induce a PCET reaction as found already for other transition metal (*e.g.* Ru) complexes.^49^,^50^ The existence of such a ligand is difficult to probe spectroscopically as the addition of a hydroxyl or aqua ligand does not change considerably the electronic configuration of the heme. Therefore, RR spectra of five coordinated and six coordinated HS–heme complexes with an aqua/hydroxyl ligand exhibit high resemblance with only minor alterations in the low frequency region from 210–450 cm^–1^.^51^,^52^ Nevertheless, hydroxyl as 6^th^ ligand has been identified in the crystal structure of the ferric FePOH and similar complexes have already been reported for a range of other heme compounds.^7^,^51^ Although OH and H_2_O as 6^th^ ligand exhibit, in general, only weak to moderate binding affinities towards the heme iron, the presence of the carboxylic acid hanging group might be able to stabilise the ligation due to hydrogen bonding interactions.^51^ In this sense, the rigid carboxylic group fixes the water/OH^–^ molecule at the heme iron cavity. A similar situation was already reported for a picket-fence Fe–heme complex in which a Fe^II^–OH_2_ was stabilized by hydrogen bonding interaction with an amide group in the 2^nd^ coordination sphere.^51^,^52^ Based on this, we propose a reaction pathway of a possible PCET reaction of the FePOH presented in Scheme 1. In this scheme, state **1**, *i.e.* ferric FePOH, carries a hydroxyl ligand that is protonated upon reduction to the ferrous state. This means, the Fe^II^–OH_2_ complex formation is achieved through 1e^–^/1H^+^ transfer. In this assumption, low pH values would afford the thermodynamic destabilisation of state **1** in favour of state **2**. This will lead to a facilitated reduction, which is consistently perceived as a positive shift of the redox potential upon lowering the pH. In the case of FePOMe, the lack of hydrogen bond interactions might result in a vacant axial position, thus, suspending a PCET reaction. Alternatively, in line with the observations, is also a scenario of ferric OH-bound FePOMe in which the hydroxyl ligand detaches upon reduction. This may also explain the subtle increase of the redox potential at lower pH, which affords lower hydroxide concentrations in solution facilitating the detachment.

**Fig. 7:**
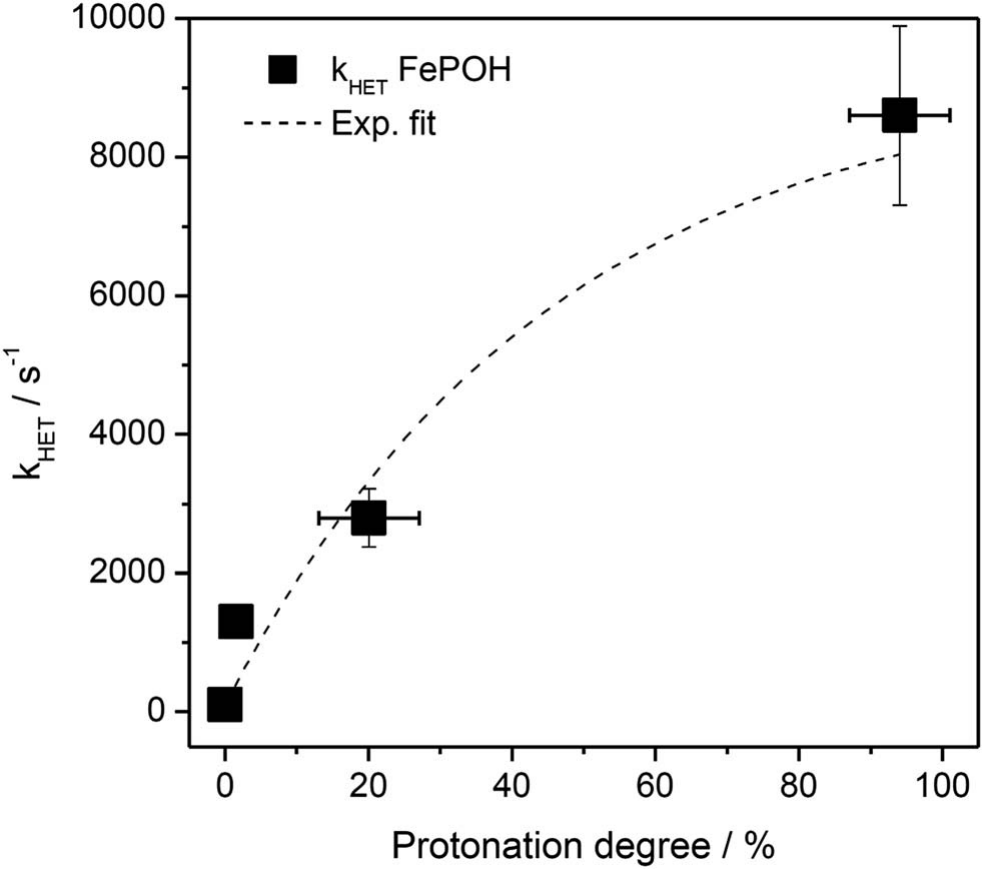
Protonation degree of the carboxylic acid hanging group of FePOH in % calculated *via* eqn (1) plotted *vs.* the TR-SERR spectroscopic derived *k*_HET_ in Table 1. The dashed line represents an exponential fit to the data.

The kinetic data obtained for the HET between electrode and the different hangman compounds supports the hypothesis. Here, FePOMe shows almost no deviation of *k*_HET_ upon changing pH and isotopic exchange. This observation is in line with both of the proposed scenarios for FePOMe above and points to a fast and unimpeded direct ET process. Moreover, the absolute rate constants lie in the range expected for direct electrode-wired heme domains and most likely involves “pure” electron tunnelling.^20^ In contrast, a distinctly different behaviour is noted for FePOH as is expected for a PCET reaction.^53^,^54^ Here, a drastic impact of the hanging group on the HET kinetics is observed. Specifically, a dependence of the HET rate on the protonation degree of the hanging carboxylic acid group was found. Fig. 7 shows the derived kinetic rate constants for FePOH from Table 1 plotted against the protonation degree calculated *via* the Henderson–Hasselbalch equation (see ESI section 7†):
1

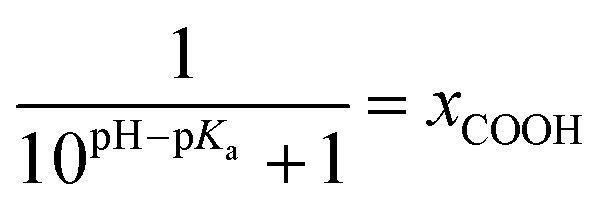




**Scheme 2:**
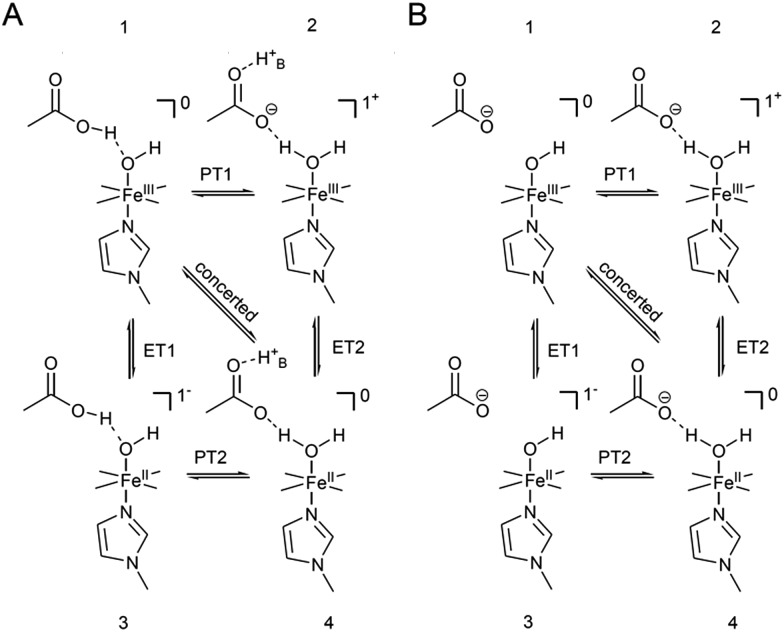
Proposed modulating interaction of the carboxylic acid hanging function in the protonated (A) and deprotonated form (B) with bound OH for FePOH. H+B denotes a proton from the bulk. Indicated charges refer only to the heme unit.

*x*_COOH_ denotes the molar fraction of the protonated carboxylic acid group at a given pH value. A clear correlation between HET rate and protonation degree can be seen in Fig. 7. The *k*_HET_ rates could be fitted reasonably with an exponential function. The general dependence of the kinetic constants on the protonation degree of the hanging group can be rationalised by considering a perturbation of the ET/PT equilibrium. In this regard, two major effects may have to be distinguished. In the simplest view, FePOH carries either a protonated acid or deprotonated carboxylate hanging function. These two different pH dependent states exhibit a different net charge resulting in an altered electrostatic environment close to the heme, and altered hydrogen bonding interactions with the bound OH/OH_2_ at the iron. Both factors are expected to exhibit a major impact on the stability/energy of the different states **1–4**. Therefore, these factors might also significantly modulate the pathway of the PCET shown in Scheme 1. Following this argumentation, the potential jump induced redox transition may also proceed differently for the two species leading to the different observed kinetic behaviour. Scheme 2 summarises the possible interactions of the protonated and deprotonated acid with the 6^th^ OH/OH_2_ ligand. Note that Scheme 2 is shown in a very minimalistic way to highlight the different reaction pathways. In principle, it cannot be excluded that an additional water molecule is placed between the 6^th^ ligand and the hanging group. This, however, does not lead to a principle change in the proposed reaction schemes. In the case of the protonated acid (Scheme 2A), formed at lower pH values, a hydrogen bond interaction between the acid function and bound OH is present that may allow efficient formation of Fe^III^–OH_2_, *i.e.* state **2***via* PT1. Hence, equilibrium between **1** and **2** is shifted to the latter, and ET may predominantly proceed *via* state **2** → **4**. In contrast, in the case of the deprotonated acid function that lacks this H-bond, direct ET1 from state **1** → **3** is rather expected (Scheme 2B). PT2 would then occur subsequent to ET by a proton from the bulk that might be pre-coordinated at the carboxylate function (not shown in the Scheme). As the TR-SERR spectroscopic experiment, however, only follows changes in the heme redox state, the water formation at the axial ligand binding site is not monitored. Comparing the two ET routes, *i.e.* ET1 and ET2, one would intuitively assume that latter is more efficient independent from the protonation state of the acid group, affording faster ET rates. In fact, ET1 involves a formation of the high energetic intermediate **3** that accommodates closely situated negative charges. This is also in line with energetic considerations that generally hold for PCET reactions.^54^ In our system, PT1 and ET1 are energetically uphill, while the corresponding transfer reactions ET2 and PT2 are downhill.^49^,^53^,^54^ Therefore, one would expect an increase of ET rate constants upon lowering the pH as ET2 becomes the dominating process. Alternatively, the stepwise ET/PT reaction might also be replaced by a concerted PCET reaction at neutral to basic pH values to proceed directly from **1** to **4** circumventing the formation of **3**. The coupling of a fast ET to a most likely slower PT process will afford significantly decreased apparent HET rate constants measured by TR-SERR spectroscopy, also in line with our experimental observations.^54^ Kinetic measurements in D_2_O can reveal the existence of a concerted PCET as the isotopic exchange would lead to a more pronounced deceleration of the HET rate constants.^54^ However, in our experiments the isotopic exchange also afforded a distinctly shifted p*K*_a_ value of the hanging group. The observed inverse KIE might therefore be rather related to an acceleration of ET rates through shift of the protonation/deuteration equilibrium in the same vein as mentioned above. Furthermore, the possible existence of a concerted PCET process, as proposed for these complexes^11^,^55^ is supported by the measurements of catalytic activity regarding H_2_O_2_ dismutation. As this reaction requires both electrons and protons, its reaction rate will be controlled by the slowest of the two charge transfer processes. At pH 7 catalytic activity of FePOH is equal or even better than for FePOMe albeit the apparent HET rate is 2 orders of magnitude lower. If a stepwise ET/PT process would be present with a constant rate for PT, the result should afford lower catalytic activity for FePOH at pH 7. In a concerted PCET process, however, the slowest reaction could be equally fast or even faster than in the case of FePOMe.

Although it is not possible to pin down unambiguously the exact reaction route, we have conclusively shown that the protonation of the hanging group is strongly influencing the HET of immobilised hangman complexes. This observation points to a strong coupling of the HET rate with the availability of protons in the 2^nd^ coordination sphere. Interestingly, such modulated ET has not been observed before in solution under non-turnover conditions. However, homogeneous reactions using an electrode as electron supplier afford slow ET rates (10^–2^ cm s^–1^).^15^ It might very well be that the influence of the 2^nd^ coordination sphere becomes only observable when high HET rates are present, which holds true for direct electrode wired complexes.^20^ This effect might be highly important for electrocatalytic efficiency of surface bound hangman complexes, and has to be investigated in the future in more detail.

## Conclusions

For the first time, the electron transfer properties of immobilised iron hangman complexes were analysed in aqueous solution *via* surface enhanced vibrational spectroscopy. The influence of a proton active hanging group in the 2^nd^ coordination sphere on the non-turnover redox thermodynamics and kinetics of the hangman complexes was studied by investigating two different hangman complexes that exhibit either an acid or an ester functionality as hanging group. Significant differences were found for these two compounds as only the acid containing complex showed a strong dependence of redox potential and heterogeneous ET kinetics on pH and H/D exchange.

Concomitantly performed SERR and SEIRA measurements were able to correlate the HET rates to the p*K*_a_ of the carboxylic acid hanging group, which was determined experimentally for the first time in aqueous buffer solution. The obtained data provides evidence for an increased HET rate with increased protonation degree of the carboxylic acid function. As possible explanation, a PCET reaction is proposed for the proton active complex that is strongly modulated by the pH dependent redox equilibrium of the hanging acid group. The overall findings shed light on the reaction mechanism of heterogenised hangman complexes in aqueous environment and demonstrate the impact of the 2^nd^ coordination sphere on the redox and kinetic properties of these catalysts immobilized on electrodes. This effect might be of high relevance for the heterogeneous catalytic activity of Fe hangman complexes or similar molecular electrocatalysts. Finally, our research proves the capability of the combination of (TR) SERR and SEIRA spectroscopy to probe 2^nd^ coordination sphere mediated reactions.

## Supplementary Material

Supplementary information
