# Supplementary material for: 2nd coordination sphere controlled electron transfer of iron hangman complexes on electrodes probed by surface enhanced vibrational spectroscopy
Source: Chem Sci. 2015 Sep 7;6(12):6999–7007. doi: 10.1039/c5sc02560e (PMC5947519; doi:10.1039/c5sc02560e)
Supplement: Supplementary file 1 [file SC-006-C5SC02560E-s001.pdf]

Electronic Supplementary Material (ESI) for Chemical Science.
This journal is © The Royal Society of Chemistry 2015

SUPPORTING INFORMATION

2nd coordination sphere controlled heterogeneous electron transfer of immobilized iron hangman complexes probed by surface enhanced vibrational spectroscopy

H. Khoa Ly, Pierre Wrzolek, Nina Heidary, Robert Götz, Marius Horch, Jacek Kozuch,

Matthias Schwalbe, Inez M. Weidinger

1. Catalytic tests

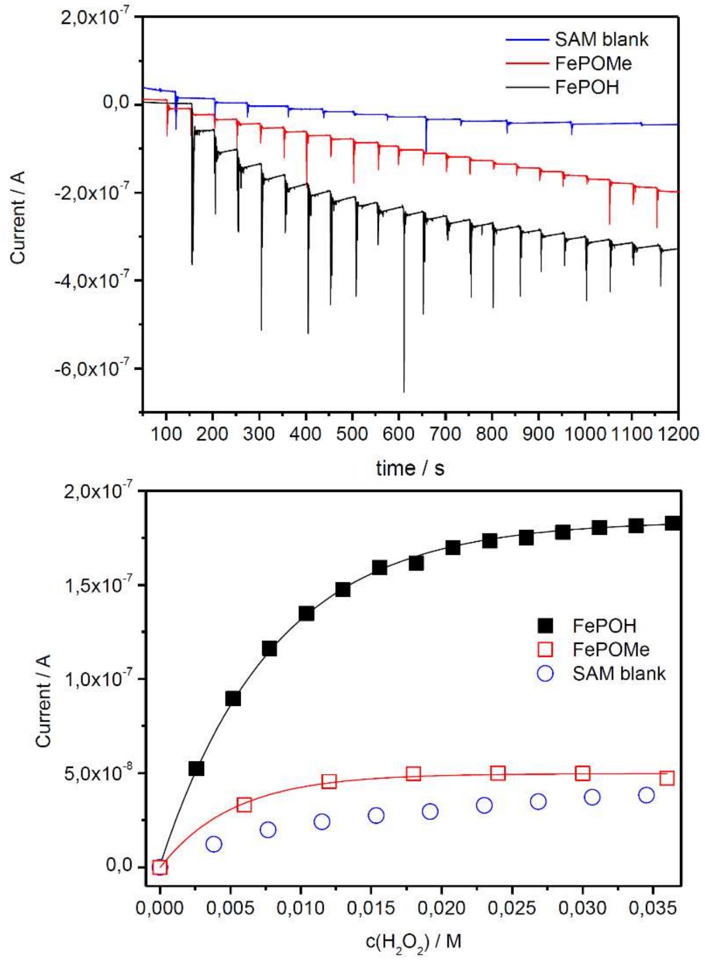

Figure S1: *Catalysis tests of adsorbed hangman compounds FePOH and FePOMe in aqueous PBS buffer at pH 7: Monitoring the catalytic current during stepwise addition of H_2O_2 (top) and plotting the (linear) corrected catalytic current vs the amount of added peroxide (bottom). Correction was achieved by subtracting the blank current.*

Catalysis tests of the adsorbed complexes were conducted using a commercial rotating Au disc electrode (Pine Instruments) with a diameter of 5 mm (geometric area: 19.6 mm²). The tests were performed in aqueous PBS buffer at pH 7. The cell volume was around 100 mL. The electrode potential was set to + 0.1 V and H_2O_2 added stepwise and the current output measured as a function of time. Current jump steps were obtained after each addition of peroxide (see Figure S1, top).[1] The saturation current represents the maximal turnover current for our systems in which the complexes are under constant turnover, and the catalytic current becomes independent from the substrate concentration (Figure S1, bottom). The turnover induced by the electrode can be derived from the blank experiment and is also shown in Figure S1. Saturation was obtained at ca. 8 ± 4 mM and 3 ± 1 mM H_2O_2 for FePOH and FePOMe, respectively. Moreover, a roughly three times higher catalytic current is observed for FePOH compared to FePOMe under otherwise identical experimental conditions.

2. Adsorption of the hangman complexes

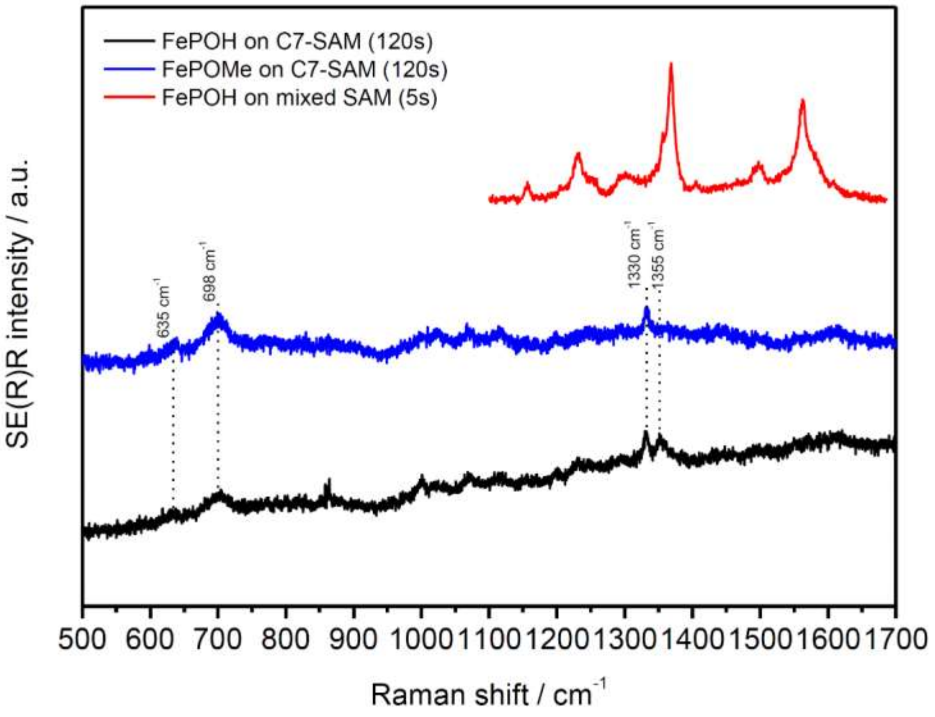

Figure S2: *SER(R) spectra of hangman complexes on rough Ag electrodes coated with C7-SH (blue and black) and mixed C7-SH/Im-SH SAM (red). In brackets, the accumulation time of the SE(R)R experiment is given.*

Adsorption of the hangman compounds was achieved by washing the electrodes with abundant EtOH, followed by ACN and DCM after SAM formation, and subsequent incubation of the electrode in ca. 10 µM solution of hangman compounds in ACN or DCM. Both solutions could be used for adsorption. After the incubation time of 2 h, the electrodes were washed with neat solvent. Adsorption tests in which a solely C7-SH coated Ag electrode was

used as substrate afforded no signals of the hangman compound in SERR experiments (see Figure S2, blue and black spectra). Using the mixed C7-SH/Im-SH monolayer, intense and well-resolved spectra of the hangman compound were obtained (Figure S2, red spectrum). Please note the different accumulation time used in the different experiments in Figure S3. The SER bands at 635 cm^{-1} and 698 cm^{-1} in the blue and black spectrum in Figure S3 can be assigned to gauche and trans ν(M-S-C) vibrations of the sulfuric monolayer.[4,5]

The formation soley of the self-assembled monolayer (SAM) on the electrode surface was monitored using SEIRA spectroscopy. Two types of SAMs were investigated, one consisting of a mix of 1-Heptanethiol (C7-SH) and 1-(11-Mercaptoundecacyl)imidazole (Im-SH) and of Im-SH solely. The Au film was incubated in an ethanolic solution containing 0.6 mM C7-SH and 0.3 mM Im-SH and 1 mM Im-SH, respectively. Figure S3 shows the resulting SEIRA spectra after the adsorption process over night (> 16 h) and cleaning with abundant EtOH using the spectra of the pure Au electrode in EtOH as a reference. Therefore, negative bands can be clearly assigned to the desorption of EtOH from the Au surface as a result of SAM formation.

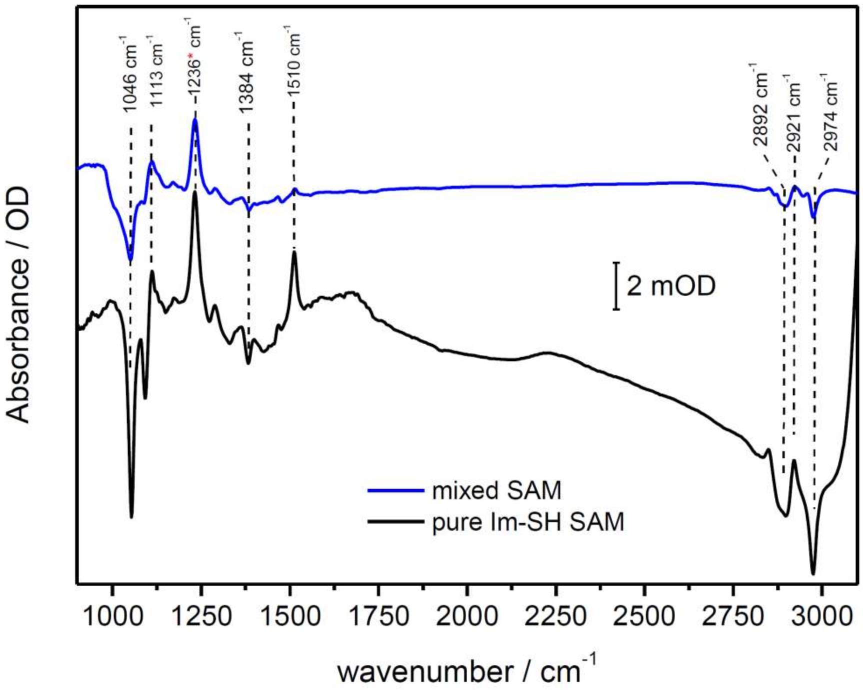

Figure S3: *Formation of mixed and pure SAM consisting of 1:2 Im-SH and C7-SH (blue) and Im-SH only (black), respectively. The band at 1236 cm^{-1} is an artefact most likely arising from temperature effects of the Si prism or traces of sulfate form the Au cleaning procedure.*

The negative bands at 1046 cm^{-1} as well as 2892 cm^{-1} and 2974 cm^{-1} originate from δ(C-H) bending and ν(C-H) stretching of the $-CH_3$ group (symmetric and asymmetric) of EtOH.[2] Positive bands indicate the adsorption of the monolayer. This comprises the bands at 1113 cm^{-1} and 2921 cm^{-1} assigned to δ(C-H) bending and ν(C-H) stretching vibrations of the $–CH_2$ groups present in the alkyl chain of the monolayer molecules.[2] Additionally, a strong band at

1510 cm^{-1} is observed. We assign this band by comparison with literature and by DFT calculations to the ν(C=C) stretching of imidazole group.[3] The fact that both SEIRA spectra do not show significant differences other than their intensity is probably based on the chemical similarity of both molecules, i.e. of C7-SH and Im-SH.

Combined, the data indicates that indeed a monolayer consisting of both C7-SH and Im-SH is formed. The hangman complexes are bound to the imidazol groups of the monolayer. The adsorption is mediated via the imidazole groups and most likely involves a coordination of the nitrogen to the heme iron as known from various other heme complexes.

3. SERR spectra evaluation and component fitting

SERR spectra of immobilized hangman compounds show typical features of resonance Raman spectra of heme containing compounds under 413 nm Soret band excitation. In the marker band region from 1300 – 1700 cm^{-1}, several prominent bands are located that can be used to track oxidation, spin and ligation state of the immobilized heme groups.[6,7] Particularly, the ν_4 band at 1355 - 1370 cm^{-1}, ν_3 band at 1495 - 1500 cm^{-1} and the ν_2 band at 1560 – 1585 cm^{-1} are indicative for electronic changes occurring at the heme iron atom.[8] In order to determine the spectral contributions of the different heme species in the SERR spectra, a component fit analysis was performed. For that, known SERR component spectra of the c-type heme protein cytochrome *c* were used as starting point and adjusted stepwise by changing band positions and relative intensities, until a set of component spectra is obtained that can be used to properly fit all hangman SERR spectra.[7,9–11] The resulting component spectra consist of a set of Lorenztian marker bands with specific band width, frequency, and relative intensities. In this respect, it was of advantage that the positions and half-width of the marker bands do not alter significantly with changed substitution pattern at the heme frame.[8] It was found that the hangman SERR spectra can be fitted satisfyingly using four component spectra by varying only a relative factor for the contributions of the different component spectra in the overall SERR spectrum (see Figures S4 and S5). Band positions, half-widths and (relative) intensities within one component spectrum were kept constant during the fitting procedure. The employed component spectra are shown in Figure S4. From the positions of their marker bands (see Table S1), the presence of a low spin (LS) and a high spin heme species (HS), each occurring in two redox states, i.e. Fe^{II} and Fe^{III}, was concluded.[6,7] Additionally to the component spectra of the heme, a component spectrum of side bands had to be included. The positions of bands in this component, however, do not interfere with the marker bands of the heme species. Figure S5 shows a so-derived complete fit for FePOH at two different potentials affording a largely oxidized (bottom) and reduced (top) hangman compound.

*Table S1: Marker band positions and SERR cross sections f_i of the four redox and conformational states of FePOH and FePOMe. * marked values were taken from literature.*[7,12]

Species	ν_4 (cm^{-1})	ν_3 (cm^{-1})	ν_2 (cm^{-1})	f_i (ν_4)
Fe^{III}-HS	1368	1494	1562	1
Fe^{II}-HS	1355	1490	1560	0.25 +/- 0.02
Fe^{III}-LS	1370	1501	1584	0.68*
Fe^{II}-LS	1360	1491	1582	0.19*

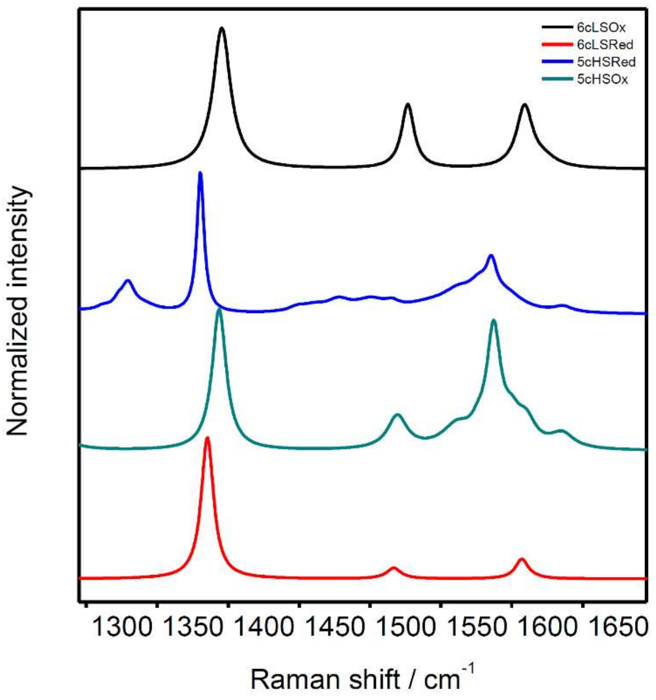

Figure S4: *Component spectra used for the spectral fitting of the hangman SERR spectra.*

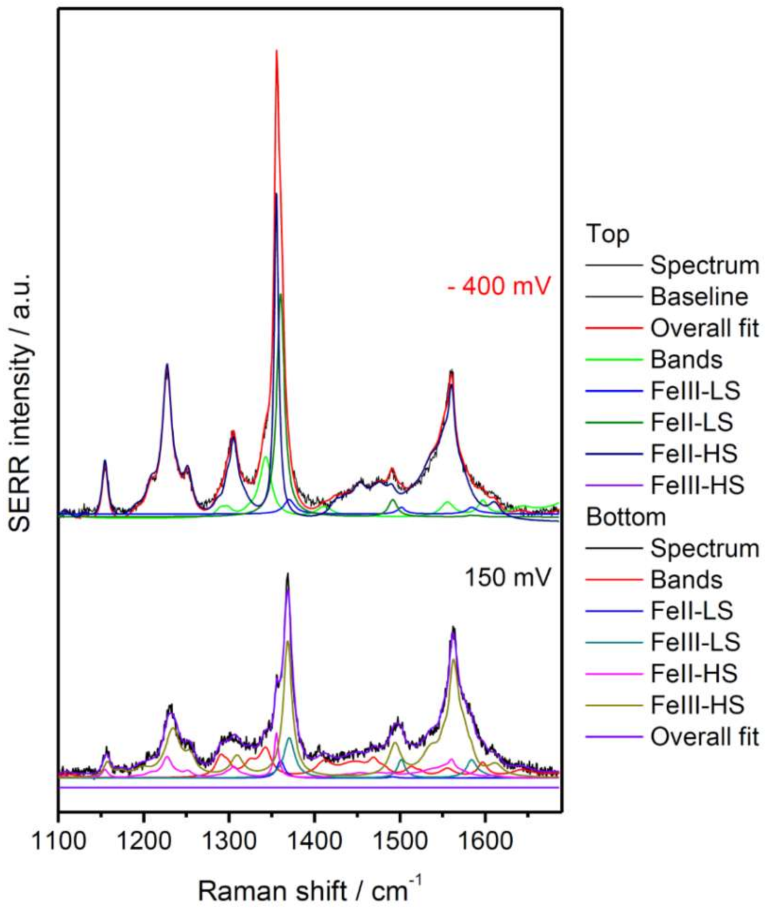

Figure S5: *SERR spectrum of FePOH at two different potentials in 100 mM PBS buffer. Overall fit and the employed five components are shown.*

4. Calculation of relative surface concentrations from SERR spectra

The relative concentrations c_i of the different surface attached heme species can be calculated using the following expression:

$$1=\sum_i c_i = \sum_i f_i \cdot I_i$$

$$\text{and } c_i = f_i \cdot I_i$$

Here, I_i denotes the intensity of the spectral contribution of the species i. f_i is the relative cross section and accounts for the different scattering probabilities of the different heme species (which is mainly due to altered resonance Raman enhancement conditions).[11,13] In our calculations, $f_{FeIII\text{-}HS}$ is set to 1 meaning that all cross sections are given relative to the scattering efficiency of the oxidized high spin component FeIII-HS. $f_{FeII\text{-}HS}$ of the respective reduced component was derived from the intensity ratio of the ν_4 band of the reduced and oxidized component spectra at +0.10V and -0.4V under otherwise same conditions, shown in Figure S6. $f_{FeII\text{-}HS}$ was calculated to be 0.25 +/- 0.02, which is in accordance with what is observed for other heme compounds in literature and accounts for the higher scattering probabilities of the reduced species as this species should have an electronic absorption maximum more close to the employed laser line.[8,13]

The cross sections accounting for the different scattering probabilities of the two spin states relative to each other, i.e. HS to LS, cannot be directly determined as no pure spectra were available. We used therefore the prior determined cross sections determined for HS and LS states of c-type hemes.[11,13,14] This is justified as we are not interested in the exact distribution of the LS vs HS component but on the chemistry of the HS state only. In this respect, a wrong value would only lead to an over- or underestimation of the presence of the low spin state with respect to the high spin state, but would not alter the derived properties from the calculations of one or the other state. Table S1 shows the employed cross sections f_i taken from respective references. The relative concentration of a species i using the listed cross sections is then calculated via

$$c_i = \frac{c_i f_i}{\sum_g c_g f_g} = \frac{c_i f_i}{0.25\, I_{FeII-HS} + 0.19\, I_{FeII-LS} + 0.68\, I_{FeIII-LS} + I_{FeIII-HS}}$$

The so-calculated relative surface concentrations of each species are shown in the manuscript in Figure 3A as a function of applied potential. The relative surface concentrations are used to determine thermodynamic and kinetic properties of the system in stationary and time-resolved SERR experiments, respectively. To extract thermodynamic properties, a Nernst equation was fitted to the data as shown in Figure 3A using the expression:

$$\left(\frac{[FeIII-HS]}{[FeII-HS]}\right)=\frac{[FeIII-HS]}{1-[FeIII-HS]}=\exp\left(\frac{nF}{RT}\left(E-E^{0}\right)\right).$$

From the inflection point of the sigmoidal shaped curve, the redox potential can be determined. Description of the kinetic analysis is provided in the manuscript or in section 6 in the supplementary information.

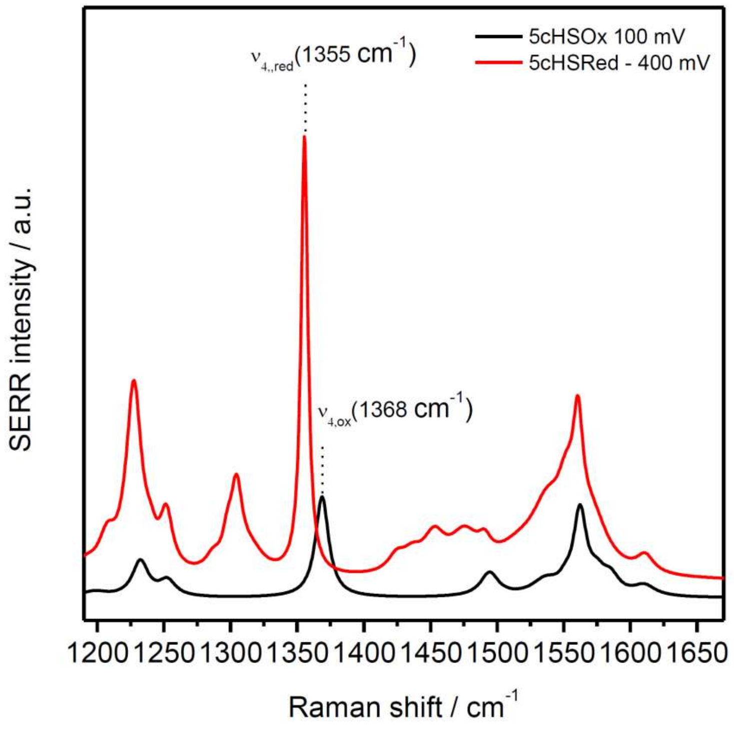

***Figure S6**: Different intensities of oxidized and reduced component spectra of FeIII-HS (black) and FeII-HS (red) at oxidizing and reducing potentials, i.e. 100 mV and – 400 mV, respectively. From the ratio, the cross section for FeII-HS is calculated.*

5. SEIRA spectroscopy

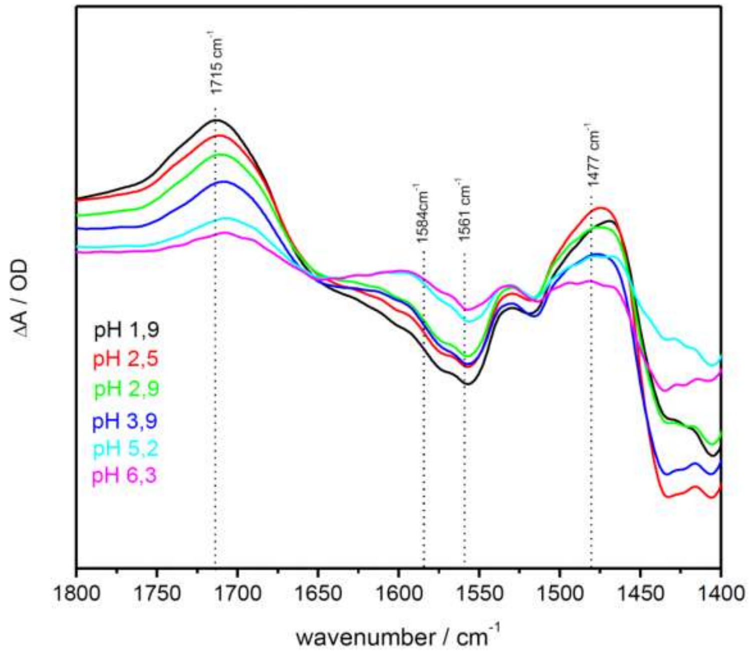

6.

Figure S7: *SEIRA difference spectra of FePOH in deuterated buffer at different pHs. Due to shift of the broad absorption of H_2O at 1640 cm^{-1}, the spectrum is much better resolved.*

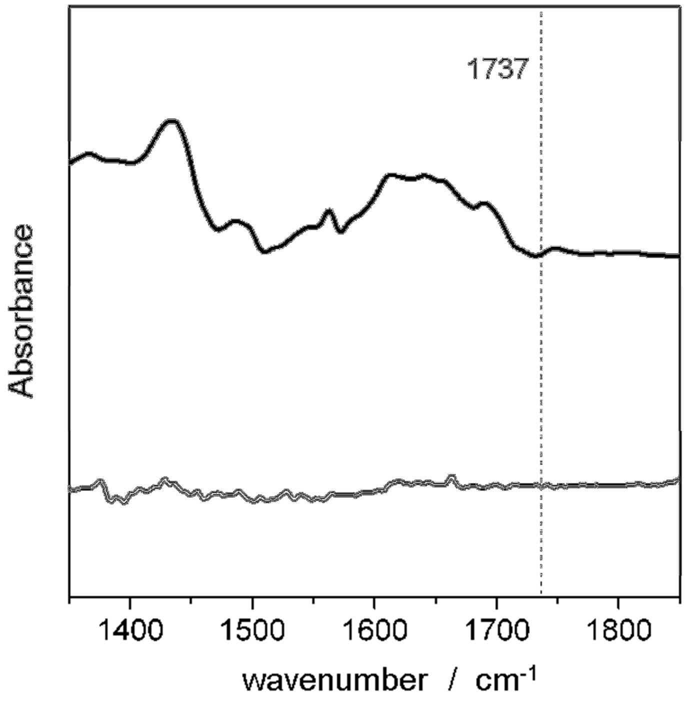

Figure S8: *Potential dependent SEIRA spectra of FePOH at 0.4 V in PBS (top) and ACN with 10 % MeOH (bottom). The reference spectra were taken at -0.2 V and 0 V, respectively.*

6. Time-resolved SERR experiments

SERR experiments were carried out in a homemade spectro-electrochemical setup. A schematic representation of the setup is shown in Figure S9 A and B. Time-resolved SERR experiments were conducted using our previously described set-up.[9,15,16] A potential jump was used to disturb the surface redox equilibrium and trigger the redox reaction. A coupling of the potential jump at the working electrode to laser light modulators (Pockell cells, Linos) using a home-made delay/pulse generator allowed recording of SERR spectra at different times after the potential jump. Figure S9 C illustrates the experimental cycle for one delay time δ. As the measurement time Δ is usually very short to afford maximal time resolution, the cycle has to be repeated several 100 times depending on the desired spectral quality and the spectra summed up together. In this respect, the recovery time RT assures that the system recovers to its initial redox state each time before inducing a new potential jump. RT and pulse time PS in our experiments have at least a ratio of 10:1 to assure full recovery. To rule out possible artefacts, e.g. hysteresis due to lack of fully redox reversibility or laser induced sample decay during the long TR experiment (usually 60 s to 120 s), the TR measurements were repeated several times thereby changing the order of measurement of the delay times and RT:PS ratio. Figure S10 shows the different TR SERR spectra for FePOH in PBS pH 4 buffer for jump from +100 mV to -250 mV recorded at different delay times δ'

The kinetic data was abstracted from the experiments by assuming first order relaxation kinetic, i.e. one-electron exchange with an electrode of a surface-adsorbed species. An exponential function was fitted to the data yielding $k_{ET} = 1 / \tau$, where τ is the time constant of the function. To account for the spectral changes occurring during the measurement interval Δ, instead of the delay time the time δ' was used with $(\delta+\Delta)/2 = \delta'$ in the plots, as shown in the manuscript in Figure 6. The probed time ranged from sub-millisecond to seconds, while only in the first milliseconds a drastic change in the surface concentrations, i.e. electron exchange, was observed. On the hundred millisecond time scale, a slower process was noted that, however, involves a much lower fraction of the redox active compounds. This behavior is exemplarily shown in Figure S11. The dashed red lines indicate the borders of the different phases. Clearly, the first phase has the highest conversion of Fe^{III}-HS (> 85 %), and the redox transition is well accounted by the exponential fit function. The origin of the second phase is not clear but due to its much low content not relevant for the kinetic analysis. Note that using a biexponential function does not improve the fitting.

.

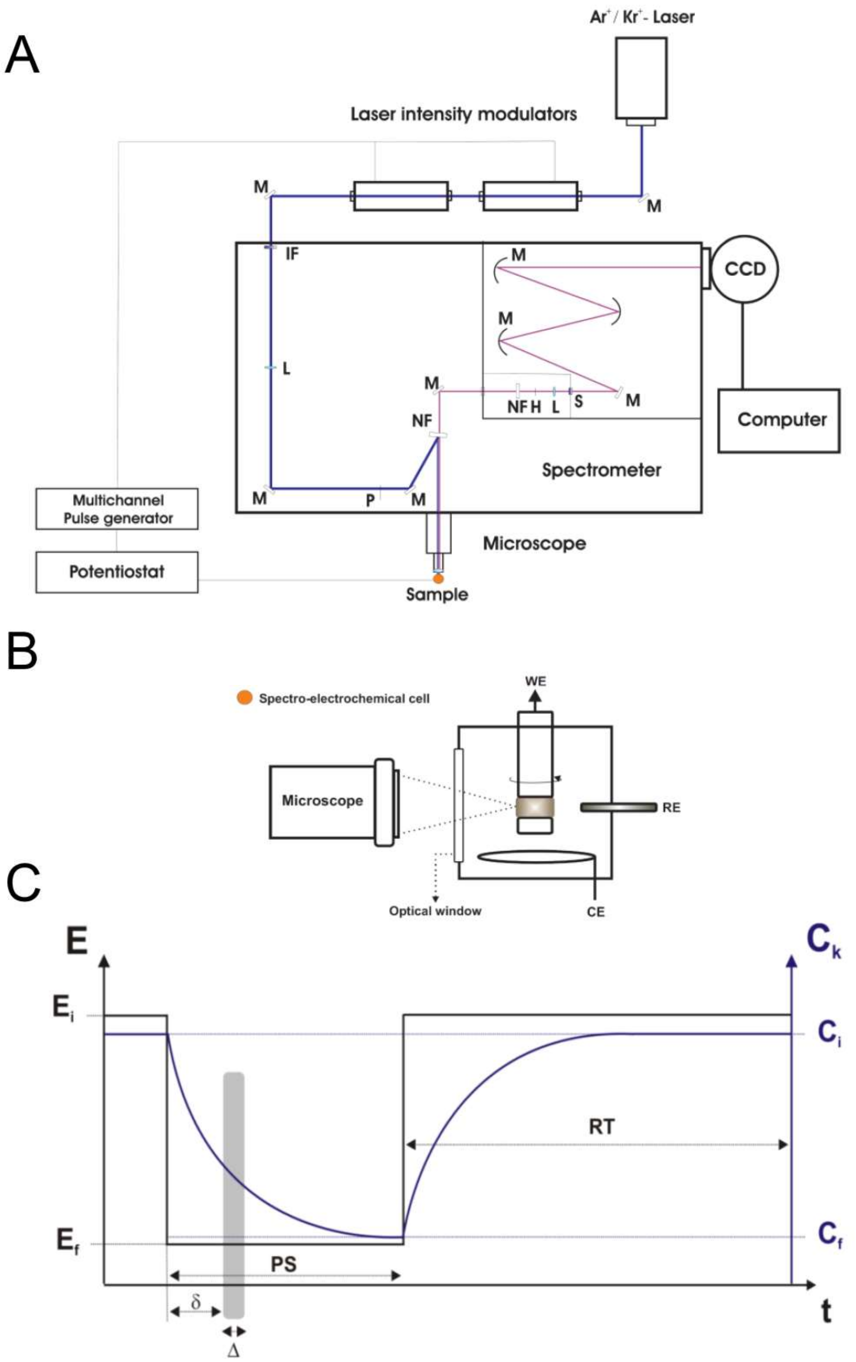

Figure S9: *A. Schematic presentation of the laser setup. CCD charged coupled display; H hole; L lens; M mirror; NF notch filter; P pinhole; S slit. B. Spectro-Electrochemical SERR setup. WE working electrode; RE reference electrode; CE counter electrode. C. TR experiment cycle for one delay time δ. Δ, PS and RT denote the measure interval, the pulse time and the recovery time, respectively. E_i/C_i and E_f/C_f represent the potential and respective surface concentration of one redox species before and after the potential jump, respectively. Figures adapted from [17].*

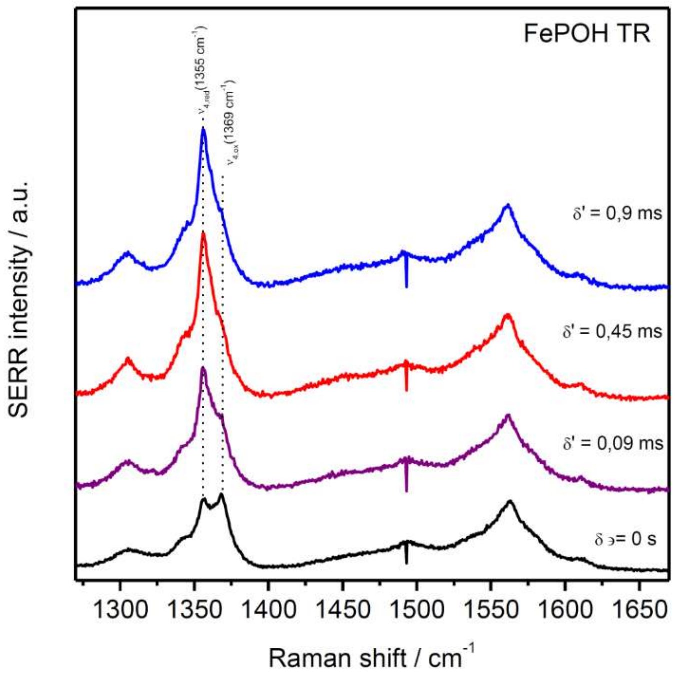

Figure S10: *TR SERR spectra of FePOH for a jump from +100 mV to - 250 mV in pH 4 PBS buffer. δ' indicates the corrected delay time between measurement and potential jump.*

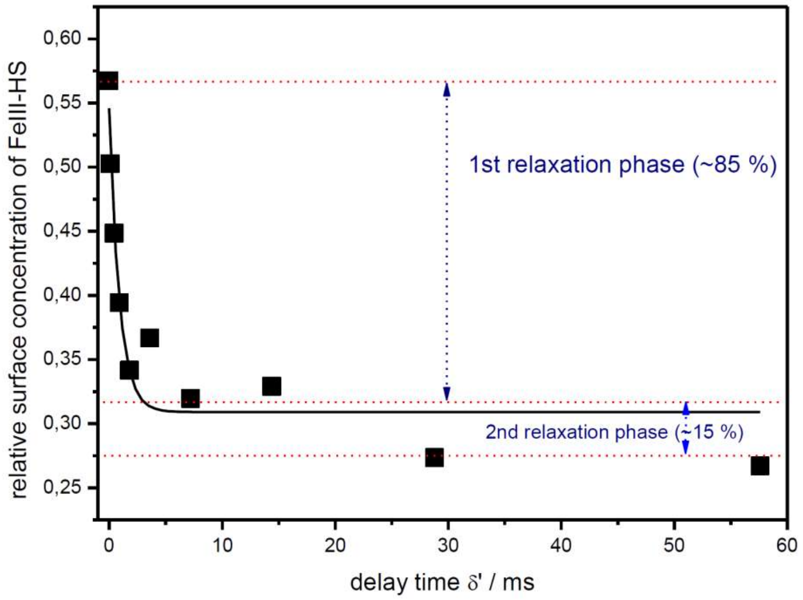

Figure S11: *Kinetic example trace of FePOH in pH 4 PBS buffer. The dashed red lines indicate the two different phases. The black line represents the exponential fit using a monoexponential function.*

7. Estimation of the protonation degree

The Henderson-Hasselbalch equation allows calculation of the protonation equilibrium of an acid as a function of the pH if the pK_a is known. This equation is used here to estimate the protonation degree of the hanging carboxylic acid group as a function of pH to derive a

correlation between HET rates and the availability of protons in the 2nd coordination sphere, i.e. at the carboxylic acid function.

$$pH = pK_a + \lg\left(\frac{[COO^-]}{[COOH]}\right)$$

$$10^{pH-pK_a} = \left(\frac{[COO^-]}{[COOH]}\right) = \left(\frac{1-x_{COOH}}{x_{COOH}}\right),$$ x_{COOH} = molar fraction

$$\frac{1}{10^{pH-pK_a}+1} = x_{COOH} \quad (1)$$ $[x_{COOH}] = [0,..,1]$

Equation (1) was used to transform the pH into protonation degree of the hanging group. The values are plotted against the obtained HET rates and shown in Figure 7 in the manuscript. The plot affords a dependence of reduction rates and protonated carboxylic acid group. Further description is found the manuscript.

Literature

1 M. Sezer, T. Genebra, S. Mendes, L. O. Martins and S. Todorovic, *Soft Matter*, 2012, **8**, 10314.

2 P. J. Larkin, *Infrared and Raman Spectroscopy*, Elsevier, 2011.

3 A. Barth, *Prog. Biophys. Mol. Biol.*, 2000, **74**, 141–173.

4 W. a Marmisolle, D. a Capdevila, E. De Llave, F. J. Williams and D. H. Murgida, *Langmuir*, 2013, **29**, 351–359.

5 M. A. Bryant and J. E. Pemberton, *J. Am. Chem. Soc.*, 1991, **113**, 3629.

6 S. Hu, I. Morris, J. Singh, K. M. Smith and T. G. Spiro, *J. Am. Chem. Soc.*, 1993, **115**, 12446–12458.

7 S. Oellerich, H. Wackerbarth and P. Hildebrandt, *J. Phys. Chem. B*, 2002, **106**, 6566–6580.

8 F. Siebert and P. Hildebrandt, *Vibrational Spectroscopy in Life Science*, Wiley-VCH, John Wiley & Sons Inc., Berlin, 1st edn., 2007.

9 D. H. Murgida and P. Hildebrandt, *Phys. Chem. Chem. Phys.*, 2005, **7**, 3773–3784.

10 H. K. Ly, N. Wisitruangsakul, M. Sezer, J. J. Feng, A. Kranich, I. M. Weidinger, I. Zebger, D. H. Murgida and P. Hildebrandt, *J. Electroanal. Chem.*, 2011, **660**, 367–376.

11 H. K. Ly, T. Utesch, I. Díaz-Moreno, J. M. García-Heredia, M. Á. De La Rosa and P. Hildebrandt, *J. Phys. Chem. B*, 2012, **116**, 5694–702.

12 H. Wackerbarth and P. Hildebrandt, *ChemPhysChem*, 2003, **4**, 714–724.

13 P. Hildebrandt and M. Stockburger, *Biochemistry*, 1989, **28**, 6710–6721.

14 H. K. Ly, N. Wisitruangsakul, M. Sezer, J.-J. Feng, A. Kranich, I. M. Weidinger, I. Zebger, D. H. Murgida and P. Hildebrandt, *J. Electroanal. Chem.*, 2011, **660**, 367–376.

15 H. Wackerbarth, U. Klar, W. Gunther and P. Hildebrandt, *Appl. Spectrosc.*, 1999, **53**, 283–291.

16 H. K. Ly, M. Sezer, N. Wisitruangsakul, J.-J. Feng, A. Kranich, D. Millo, I. M. Weidinger, I. Zebger, D. H. Murgida and P. Hildebrandt, *FEBS J.*, 2011, **278**, 1382–90.

17 K. H. Ly, PhD Thesis, Technische Universität Berlin, 2012.
